# Evaluation of the efficacy of drug treatment based on measurement of the oxidative stress, using reactive oxygen metabolites and biological antioxidant potential, in children with autism spectrum disorder and attention deficit hyperactivity disorder

**DOI:** 10.1186/s40780-020-00164-w

**Published:** 2020-04-25

**Authors:** Taisuke Kitaoka, Masahito Morimoto, Toshiaki Hashimoto, Yoshimi Tsuda, Tadanori Nakatsu, Shojiro Kyotani

**Affiliations:** 1 Tokushima Bunri University, Graduate School of Pharmaceutical Sciences, Nishihamahouji, Yamashiro-cho, Tokushima-shi, Tokushima, 770-8514 Japan; 2 Department of pharmacy, Japanese Red Cross Tokushima Hinomine Rehabilitation Center for People with Disabilities, Shinbiraki, Chuden-cho, Komathushima-shi, Tokushima, 773-0014 Japan; 3 Department of pediatrics, Japanese Red Cross Tokushima Hinomine Rehabilitation Center for People with Disabilities, Shinbiraki, Chuden-cho, Komathushima-shi, Tokushima, 773-0014 Japan

**Keywords:** Pediatrics, Oxidative stress, Autism spectrum disorder, Attention deficit hyperactivity disorder, Reactive oxygen metabolites, Objective assessment marker

## Abstract

**Background:**

Autism spectrum disorder (ASD) is a neurodevelopmental disorder, mainly characterized by impairment of social communication and restricted interests. ASD is frequently accompanied by attention deficit hyperactivity disorder (ADHD), which is characterized by carelessness, hyperactivity and impulsivity (ASD/ADHD). It has been suggested that ASD and ADHD are associated with oxidative stress, that is, that patients with ASD/ADHD are in a state of increased oxidative stress. There are currenr tly no objective or biological test criteria for evaluating the efficacy of drug therapy in these patients. The purpose of this study was to evaluate whether oxidative stress markers [serum reactive oxygen metabolites (d-ROMs) levels and biological antioxidant potential (BAP)] can be used as objective indicators for evaluating the efficacy of drug treatment in ASD/ADHD patients.

**Methods:**

The subjects of this study subjects were 50 Japanese patients with ASD/ADHD aged 4 to 14 years old. Serum samples were obtained from the patients to measure the serum levels of d-ROMs and the serum BAP. The study subjects were divided into two age groups: preschool children (4 to 6 years old) and school-age children (7 to 14 years old), and the serum levels of d-ROMs, serum BAP, serum BAP/d-ROMs ratio (hereinafter, the prefix serum will be dropped), and scores on the Parent-interview ASD Rating Scales-Text Revision (PARS-TR) and ADHD Rating Scale (ADHD-RS) were determined before and after drug therapy and compared between the two groups. In addition, changes in the d-ROMs, BAP and BAP/d-ROMs ratio and changes in the scores on the PARS-TR and ADHD-RS after treatment were also analyzed.

**Results:**

Significant decrease of the d-ROMs, BAP, and scores on the PARS-TR and ADHD-RS, with a significant increase of the BAP/d-ROMs ratio, was observed after treatment. In addition, a significant correlation was observed between the changes in the d-ROMs and changes in the scores on the PARS-TR and ADHD-RS after treatment in the school-age ASD/ADHD children.

**Conclusion:**

Our results suggest the possibility that the serum level of d-ROMs may be useful as an objective assessment marker to supplement the subjective assessment of the effects of drug treatment in school-age children with ASD/ADHD.

## Background

Autism spectrum disorder (ASD) is a neurodevelopmental disorder, mainly characterized by impairments in social communication and interpersonal interactions, restricted interests, and repetitive behaviors. In the 5th version of the Diagnostic and Statistical Manual of Mental Disorders (DSM-5) [1], revised in 2013, the diagnostic label was changed from pervasive developmental disorder (PDD) to ASD. The DSM-5 formally recognizes the coexistence (ASD/ADHD) of ASD and attention deficit hyperactivity disorder (ADHD), which is characterized by carelessness, hyperactivity and impulsivity [1]. In recent years, the prevalence of ASD/ADHD has been on the rise [2], and it has been reported that approximately 80% of ASD patients who visit medical institutions are concomitantly diagnosed as also having ADHD [3]. Patients with ASD/ADHD, as compared with those with ASD or ADHD alone, have been reported to show lower adaptability and a higher degree of stress in daily life, and to have a poorer quality of life [4, 5]. In addition, it has also been reported that the risk of secondary disorders, such as anxiety and mood disorders, is elevated in patients with ASD/ADHD [6]. Therefore, drug therapy is selected depending on the individual patient’s condition; however, there are no objective or biological test criteria to evaluate the efficacy of drug therapy in these patients.

Neurodevelopmental disorders such as ASD and ADHD have been suggested to be associated with oxidative stress [7–10] and many oxidative stress markers such as superoxide dismutase, nitric oxide synthase, xanthine oxidase, glutathione S-transferase and paraoxonase-1 have been reported [7, 8, 10, 11]. However, these markers cannot be measured immediately in clinical practice because of the need for specialized equipment and cannot be used comprehensively to assess stress in vivo. On the other hand, d-ROMs and BAP are regarded as comprehensive biological markers of stress [12]. Furthermore, the measurement can be performed using the same sample and test equipment, and the measurement method is a simple procedure in about 5 min for each test. For these reasons we focused on d-ROMs and BAP. We proposed that these oxidative stress markers could be used as objective indicators in the assessment of stress in children [13]. The aim of this study was to investigate whether the serum levels of d-ROMs and the serum BAP, as oxidative stress markers, can be used as objective indicators for evaluating the efficacy of drug treatment in children with ASD/ADHD.

## Methods

### Study subjects

The study subjects were 50 Japanese patients (38 males and 12 females) with ASD/ADHD aged 4 to 14 years old, who had never received any drug treatment intervention. The eligibility criteria for inclusion in the study were patients diagnosed as having ASD/ADHD according to DSM-5-based assessment and clinical symptoms documented by a pediatric neurologist. Patients with underlying diseases other than developmental disorders, and those taking drugs for purposes other than the treatment of ASD or ADHD or than improvement in the symptoms were excluded. In our previous study, younger children had higher oxidative stress levels [13]. And it has been reported that the stress level of children differs due to changes in the environment between preschool and school life [14]. In consideration of the impact of age and stress from the living environment, the study subjects were divided into two groups by age: preschool children (4 to 6 years old) and school-age children (7 to 14 years old).

### Measurement items

#### Indicator of oxidative stress (d-ROMs test)

The d-ROMs test is used to measure the serum levels of hydroperoxide. Hydroperoxide is a metabolite generated when proteins, lipids, amino acids, nucleotides, etc., are oxidized by active oxygen or free radicals, and generates free radicals in the presence of metal ions. Therefore, measurement of the hydroperoxide levels in the serum reflects the degree of biological oxidative stress. U.CARR is used for the unit, and 1 U.CARR is equivalent to the H_2_O_2_ of 0.08 mg/dL.

#### Indicator of antioxidant capacity (BAP test)

The BAP test is used to measure the degree to which ferric iron can be reduced to ferrous iron by the antioxidants contained in the blood. Measurement of the reducing ability as a blocking effect against the peroxidation chain reaction induced by reactive oxygen species or free radicals allows assessment of the antioxidant capacity, which is the ability to scavenge free radicals in the body. Μmol/L is used for the unit.

### Measurement method

Between November 2016 and September 2019, blood samples were collected from the subjects prior to the start of treatment. Within one year after the start of the drug treatment, blood samples were collected again. Blood samples were centrifuged at 1469 g for 10 min, to obtain serum samples. The d-ROMs and BAP were measured using FREE CARRIO DUO (Diacron International, Grosseto, Italy), a free radical analyzer.

#### d-ROMs

First, each serum sample (20 μL) was placed in a cuvette filled with a pH 4.8-buffer that had been warmed in an incubator for at least 15 min. Then, the cuvette was gently overturned and mixed to allow Fe2^+^ and Fe3^+^ separation from the blood proteins. Fe2^+^ and Fe3^+^ act as catalyzers, and result in the degradation of blood hydroperoxide into an alkoxy radical and a peroxy radical. N,N-diethyl-p-phenylenediamine (20 μL) was then placed in the cuvette as a color-developing chromogen, resulting in the oxidation of the chromogen substrate by the free radicals to yield a red-colored radical cation. The cuvette was again overturned and mixed, then placed in a spectrophotometer to measure the radical cations at 505 nm.

#### BAP

50 μL of a chromogenic reagent (containing Fe3^+^) for BAP was added to a cuvette to induce red coloration, and the contents were mixed by inversion of the cuvette, followed by spectrophotometric measurement of the coloration. Then, each serum sample (10 μL) was placed into the cuvette and mixed. The cuvette was then placed in a thermostat to allow reaction in the mixture. Finally, the cuvette was placed in a spectrophotometer for measurements at 505 nm.

### Investigation items

#### Parent-interview ASD rating scales–text. Revision (PARS-TR)

PARS-TR is a rating scale used to assess the presence or absence of developmental and behavioral symptoms of ASD and their severity, through interviewing the mothers (or other primary caregivers, if it is difficult to obtain information from the mothers) of the patients. In this study, a pediatric neurologist scored the rating scale. The minimum eligible age for assessment by PARS-TR is 3 years. The scale consists of 57 items classified into 6 subscales: interpersonal relations, communication, obsession, stereotyped behaviors, difficulty in living, and hypersensitivity (34 items for preschoolers, 33 items for school-age children, and 33 items for adolescence and adulthood). The severity of each item is rated on a 3-point scale (0, 1 and 2). The higher the total score, the more likely that the assessee child has ASD.

#### ADHD rating scale (ADHD-RS)

ADHD-RS is a rating scale used to assess the presence or absence of ADHD symptoms and their severity through interviewing people around the patients. In this assessment, information is obtained from the mothers (or other primary caregivers, if it is difficult to obtain information from the mothers) of the subjects. In this study, a pediatric neurologist scored the rating scale. The minimum eligible age for assessment by ADHD-RS is 5 years. The scale consists of 18 items in total: 9 items related to inattention and 9 items related to hyperactivity/impulsivity. The severity of each item is evaluated on a 4-point scale (0, 1, 2 and 3). The higher the total score, the more likely the assessee child has ADHD.

### Statistical analysis

The d-ROMs, BAP and BAP/d-ROMs ratio, and the scores on the PARS-TR and ADHD-RS were compared before and after drug treatment using Wilcoxon signed-rank test, with the level of significance (*p* value) set at 0.05. In addition, the Pearson correlation coefficient (r) and *p*-values were calculated, with the level of significance set at 0.05, to examine the correlations between the pre- and post-treatment changes in the d-ROMs, BAP and BAP/d-ROMs ratio and the pre- and post-treatment changes in the scores on the PARS-TR and ADHD-RS. The statistical analysis software used was R (version 3.6.1).

## Results

**Fig. 1:**
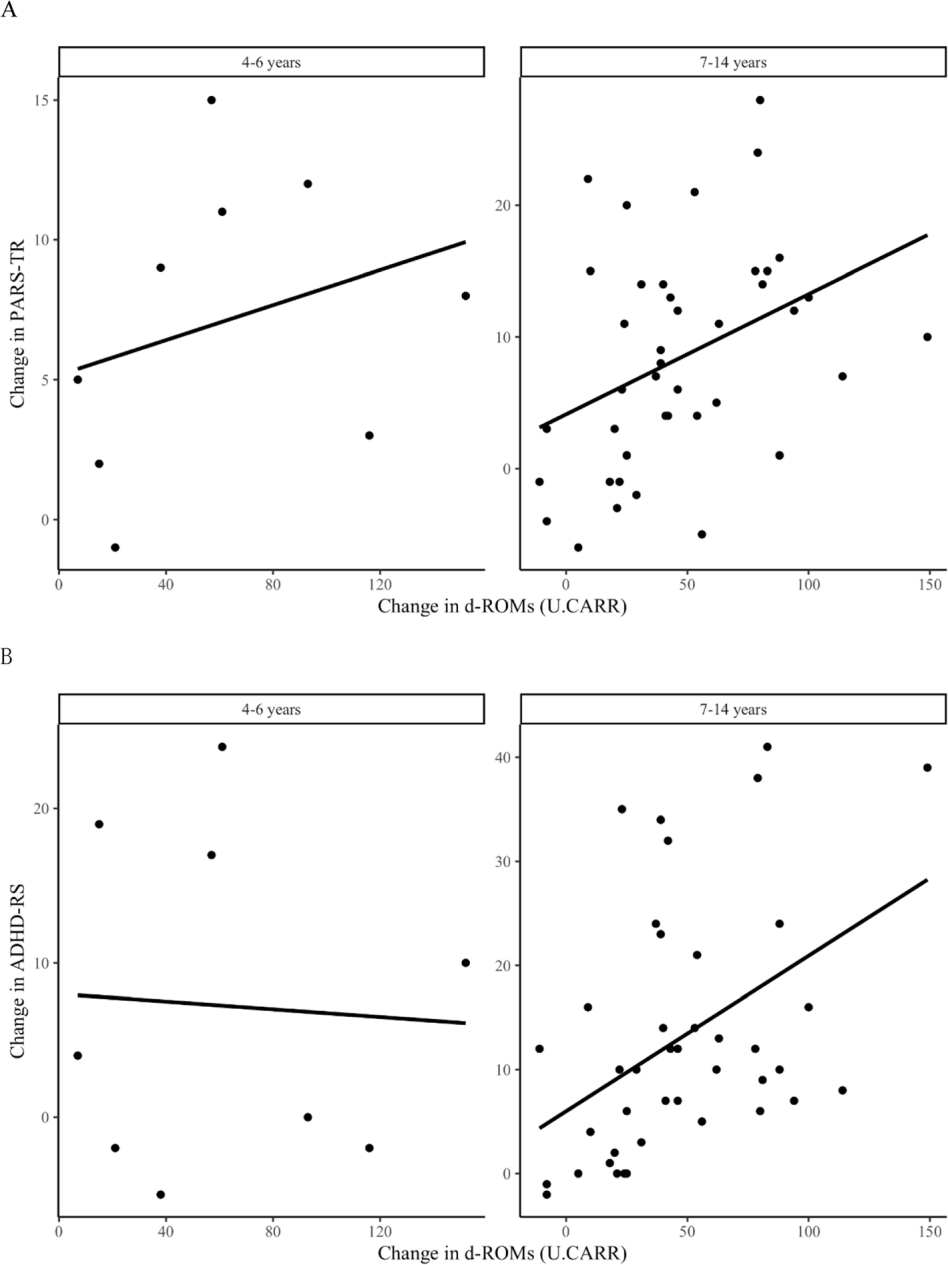
Relationship between change in PARS-TR and ADHD-RS scores and change in d-ROMs levels. 1A = Relationship between change in PARS-TR score and change in d-ROMs levels. In the 7-14 years group, there was a significant correlation between the change in the d-ROMs value and the change in the PARS-TR score. (*r* = 0.39, *p* = 0.01), but not in the 4–6 years group. 1B = Relationship between change in ADHD-RS score and change in d-ROMs levels. In the 7-14 years group, there was a significant correlation between the change in the d-ROMs value and the change in the ADHD-RS score. (*r* = 0.44, *p* = 0.004), but not in the 4–6 years group

Table 1 shows the demographic characteristics of the study subjects. The mean age was 8.64 ± 2.46 years, with the minimum and maximum ages of 4 and 14 years. In regard to the sex distribution, males accounted for 76.0% of all the study subjects, and also for the majority of subjects in both the groups of preschool and school-age children. The number of treatment drugs used in the study subjects was 11 for ASD/ADHD, and the most commonly prescribed drug was guanfacine hydrochloride, which was used in a half of all the subjects. Table 2 shows the pre- to post-treatment changes in each of the measurement items in the study subjects. Significant decreases of the d-ROMs and BAP, and in the scores on the PARS-TR and ADHD-RS, with a significant increase in the BAP/d-ROMs ratio, were observed in all the subjects after treatment. The preschool children showed a significant decrease of both the d-ROMs and the score on the PARS-TR after treatment. On the other hand, the school-age children showed significant decreases of the d-ROMs, BAP and the scores on the PARS-TR and ADHD-RS, with a significant increase of the BAP/d-ROMs ratio, after treatment. Table 3 shows the relationships between the pre- to post-treatment changes in the d-ROMs, BAP and BAP/d-ROMs ratio, and the pre- to post-treatment changes in the scores on the PARS-TR and ADHD-RS. Significant correlations were observed in all the subjects between the pre- to post-treatment changes in the d-ROMs and the pre- to post-treatment changes in the scores on the PARS-TR (*r* = 0.34, *P* = 0.02) and ADHD-RS (*r* = 0.29, *P* = 0.04). Figure 1 shows the relationship between the pre- to post-treatment changes in the serum d-ROMs and the pre- to post-treatment changes in the scores on the PARS-TR and ADHD-RS in the preschool and school-age children. In the school-age children, significant correlations were observed between the pre- to post-treatment change in the d-ROMs and pre- to post-treatment change in the scores on the PARS-TR (*r* = 0.39, *P* = 0.01) and ADHD-RS (*r* = 0.44, *P* = 0.004). On the other hand, in the preschool children, no significant correlations were observed for any of the items.

**Table 1:** Background of subjects

Age(y)	
Means ± SD	8.62 ± 2.46
Median	8
Maximum	14
Minimum	4
Sex	
Male	38 (76.0%)
Female	12 (24.0%)
Gender comparison by age group	
4–6 y (*n* = 9)	
Male	6 (66.7%)
Female	3 (33.3%)
7–14 y (*n* = 41)	
Male	32 (78.0%)
Female	9 (22.0%)
Drug^a^	
Guanfacine Hydrochloride	25 (50.0%)
Methylphenidate Hydrochloride	18 (36.0%)
Risperidone	12 (24.0%)
Aripiprazole	11 (22.0%)
Atomoxetine Hydrochloride	9 (18.0%)
Ramelteon	2 (4%)
Hangekobokuto (Kampo medicine)	2 (4%)
Yokukansan (Kampo medicine)	2 (4%)
Yokukansankachimpihange (Kampo medicine)	1 (2%)
Sertraline Hydrochloride	1 (2%)
Sodium Valproate	1 (2%)

*SD* Standard deviation y = years

^a^ Each of these drugs may also be used in combination

**Table 2:** The pre- to post-treatment changes in objective markers and subjective scores

All subjects	Before	After	Change^a^	p ^b^
d-ROMs(U.CARR)	413.1	363.3	49.8	<.001
BAP (μmol/L)	2521.5	2403.3	118.2	0.007
BAP/d-ROMs	6.16	6.65	−0.50	<.001
ADHD-RS	27.4	15.40	12.00	<.001
PARS-TR	21.3	13.2	8.18	<.001
**4–6 years**				
d-ROMs(U.CARR)	443.8	381.6	62.2	0.004
BAP (μmol/L)	2679.6	2467.4	212.1	0.05
BAP/d-ROMs	6.13	6.51	−0.38	0.13
ADHD-RS	24.7	17.4	7.22	0.14
PARS-TR	19.4	12.3	7.11	0.008
**7–14 years**				
d-ROMs(U.CARR)	406.3	359.2	47.1	<.001
BAP (μmol/L)	2486.8	23,891.2	97.6	0.03
BAP/d-ROMs	6.16	6.68	−0.52	<.001
ADHD-RS	28.0	15.0	13.00	<.001
PARS-TR	21.7	13.3	8.4	<.001

^a^Change is the score obtained by subtracting the score after treatment from the score before treatment

^b^ Wilcoxon signed-rank test was used to compute *p*-values

**Table 3:** Correlation between change in objective markers and changes in subjective scores

	ADHD-RS		PARS-TR	
All subjects	r^a^	p	r	p
d-ROMs(U.CARR)	0.29	0.04	0.34	0.02
BAP (μmol/L)	0.09	0.51	0.17	0.24
BAP/d-ROMs	−0.14	0.32	−0.11	0.46
**4–6 years**				
d-ROMs(U.CARR)	−0.06	0.88	0.30	0.44
BAP (μmol/L)	−0.11	0.78	0.05	0.89
BAP/d-ROMs	−0.12	0.77	−0.23	0.55
**7–14 years**				
d-ROMs(U.CARR)	0.44	0.004	0.39	0.01
BAP (μmol/L)	0.16	0.31	0.19	0.22
BAP/d-ROMs	−0.14	0.39	−0.09	0.58

^a^ Pearson correlation coefficient

## Discussion

The reported male-to-female ratio in childhood ADHD varies in the range of 2:1 to 10:1 [15, 16] and that of childhood ASD is 4:1 [17, 18]. About 80% of the subjects in the present study were males. A previous epidemiological study also reported a higher prevalence of developmental disorders in males [19]. Although the impact of gender differences is an important factor to consider, Nagata et al. [20] reported that there are no gender differences in the d-ROMs or BAP. We have also confirmed the absence of any gender differences in these parameters in our previous studies [13]. In addition, in our previous study, the d-ROMs level was significantly higher in group A (3 to 6 years old) than in group B (7 to 11 years old) or group C (12 to 15 years old) (A vs. B: *p* < 0.001, A vs. C: p < 0.001) [13]. And it has been reported that changes in the environment between preschool and school life lead to different levels of stress in children [14]. Similar tendency was also observed in the present study; d-ROMs are higher in preschool children than school-age children with ASD/ADHD. These findings are speculated to be associated with rapid synaptic pruning and regeneration in childhood [21, 22]. In addition, they may also be affected by psychological stress from anxiety and fear, as preschool children have more anxiety and fear of blood collection than school-age children.

ASD is often accompanied by emotional disorders, including anxiety disorders, mood disorders, and phobias due to psychological stress [23]. Therefore, ASD patients are more likely to exhibit problematic behaviors, such as aggressive behaviors, self-harm, tantrums and insomnia [24]. In the present study also, some subjects were on treatment with sodium valproate or ramelteon for these secondary disorders. In addition, enhanced oxidative stress, caused by mitochondrial dysfunction and environmental factors, has been reported to be a factor involved in the brain damage in ASD patients. ASD patients are considered to be in a high oxidative stress state [7, 8]. On the other hand, an imbalance between oxidative and anti-oxidative processes and enhanced oxidative stress have also been reported in ADHD patients [9, 10]. Taken together, ASD/ADHD patients are considered to have high degrees of oxidative stress and decreased antioxidant capacity. This can also be inferred from comparisons with oxidative stress marker data from healthy children obtained in our previous study [13], but we cannot directly compare with results in our previous study and results in the present study. A significant decrease in the d-ROMs and significant increase in the BAP/d-ROMs ratio were observed after treatment in the present study. The PARS-TR and ADHD-RS, used as assessment tools in the present study, are subjective indicators, based on observations. In the present study, the scores on both PARS-TR and ADHD-RS decreased significantly after treatment. These results suggest that, drug therapy-induced behavioral changes and improvement in symptoms have the effect of reducing the psychological stress in daily life, thereby leading to changes in the d-ROMs and BAP/d-ROMs ratio. In addition, in the present study, besides the study subjects being given drug therapy, both the study subjects and their caregivers were also provided with psychological and educational guidance at the time of their hospital visits. Therefore, the possibility that the nursing care guidance provided to the patients and their caregivers may also have contributed to the reduction in the psychological stress in daily life, leading to changes in the d-ROMs and BAP/d-ROMs ratio, cannot be excluded.

Atomoxetine hydrochloride, which was prescribed for 18.0% of the study subjects, has been reported to cause mitochondrial dysfunction when used at high concentrations [25]. Therefore, it is considered highly necessary to assess oxidative stress using objective biomarkers for selecting the appropriate drug dose.

In the present study, significant correlations were observed between the pre- to post-treatment changes in the d-ROMs and the pre- to post-treatment changes in the scores on the PARS-TR and ADHD-RS, suggesting the existence of a correlation between the subjective and objective indicators.

The efficacy and safety have not been established of guanfacine hydrochloride, atomoxetine hydrochloride and methylphenidate hydrochloride in ADHD children aged less than 6 years old, of aripiprazole in ASD children aged less than 6 years old, and of risperidone in ASD children aged less than 5 years old. Therefore, use of these drugs in the respective age groups represents off-label use in Japan. Thus, there is a lack of evidence for drug therapy in preschool children with ASD/ADHD. In the present study, in consideration of the impact of age and stress from the living environment, such as the school, the study subjects were classified into two age groups: preschool children and school-age children. However, there was no pre- to post-treatment change in the score on either PARS-TR or ADHD-RS in the preschool children, which may be attributed to the possible need for use of different cutoff values of the PARS-TR score in preschool and school-age children [26] and also the difficulty in assessing the ADHD-RS score in 4-year-old children because the minimum age eligible for assessment by ADHD-RS is 5 years.

The present study had several limitations. First, since it was impossible to conduct a detailed investigation of the interpersonal relations and living environment of the study subjects, the potential impact of these factors on the study results could not be verified. Secondly, there are many patients in clinical practice who are followed up with only psychological and educational guidance, etc., without drug therapy. It was not possible to collect blood samples from such patients who received only nursing care guidance, and only subjects who received drug treatment were enrolled in the study. Therefore, as a future issue, it is necessary to verify and compare the results between patients receiving drug therapy with nursing care guidance and those receiving nursing care guidance only, in order to demonstrate the usefulness of drug therapy. Thirdly, the sample size was not sufficient. In particular, the number of preschool children was small. Furthermore, many combinations of drugs were used in the study subjects, making it impossible to assess the oxidative stress markers for each drug. Therefore, in the future, it is necessary to examine the impact of each drug on the measured oxidative stress markers and to carry out re-validation in preschool children by further increasing the sample size.

## Conclusions

In the present study, the d-ROMs and the scores on the PARS-TR and ADHD-RS decreased significantly after drug treatment. In addition, in school-age children with ASD/ADHD, significant correlations were observed between the pre- to post-treatment changes in the d-ROMs and the pre- to post-treatment changes in the scores on the PARS-TR and ADHD-RS. These findings suggest that the serum level of d-ROMs, an oxidative stress marker, may be a useful objective marker to assess effects of drug therapy in school-age children with ASD/ADHD.

## Data Availability

All data generated or analyzed in this study are included in this article.

## Abbreviations

ASD: Autism spectrum disorder
ADHD: Attention deficit hyperactivity disorder
d-ROMs: Reactive oxygen metabolites
BAP: Biological antioxidant potential
PARS-TR: Parent-interview autism spectrum disorder rating scales-Text Revision
ADHD-RS: Attention deficit hyperactivity disorder- rating scale
DSM-5: 5th version of the diagnostic and statistical manual of mental disorders
PDD: Pervasive developmental disorder
